# Pretreating Recycled Carbon Fiber Nonwoven with a Sizing Formulation to Improve the Performance of Thermoplastic Recycled Fiber-Reinforced Composites

**DOI:** 10.3390/polym16040561

**Published:** 2024-02-19

**Authors:** Frederik Goethals, Elke Demeyer, Isabel De Schrijver, Myriam Vanneste

**Affiliations:** 1 Centexbel-Zwijnaarde, Centexbel-VKC, 9052 Zwijnaarde, Belgium; ids@centexbel.be (I.D.S.); mv@centexbel.be (M.V.); 2 VKC, Centexbel-VKC, 8500 Kortrijk, Belgium; edm@vkc.be

**Keywords:** recycled carbon fiber, fiber sizing, nonwoven, thermoplastic composites, polyamide, polypropylene, spray

## Abstract

Pyrolysis is already an established recycling method to recover the carbon fibers of end-of-life composites. However, the pyrolysis process removes the fiber sizing. Fiber sizing is a critical step in composite material production, influencing adhesion, protection and overall performance. In this study, recycled carbon nonwoven reinforcements made from pyrolyzed carbon fibers were pretreated to improve the mechanical properties of polyamide and polypropylene composites. The pretreatment involved applying specific coatings (sizings) on the nonwoven by spraying. Pretreated and non-pretreated composites were prepared by compression molding to investigate the impact of the fiber pretreatment on the tensile properties and interlaminar shear strength. The tests were performed in the 0° and 90° directions of the composite plate. The results revealed that pretreatment had little effect on the polyamide composites. However, significant improvements were obtained for the polypropylene composites, as an increase of more than 50% in tensile strength was achieved in the 0° direction and more than 35% in the 90° direction. In addition, the interlaminar shear strength increased from 11.9 MPa to 14.3 MPa in the 0° direction and from 14.9 MPa to 17.8 MPa in the 90° direction.

## 1. Introduction

One of the most used fibers in composite materials is carbon fiber. This fiber has excellent properties like light weight, high stiffness, high mechanical strength, high dimensional stability and high corrosion resistance [1]. These properties, in combination with the high price of carbon fibers, make it worthwhile to recycle these fibers [2]. With the recycled carbon fibers, new composites can be produced, and some application examples are oil pans, seat backs, chassis panels and structural panels [3]. 

Carbon fibers can be recycled from post-industrial waste, which includes dry carbon waste and prepreg scrap, and from end-of-life composites [4]. The recycling of dry post-industrial waste mainly involves chopping leftovers from yarns, filaments and fabrics into short fibers. In this case, recycled carbon fibers basically have the same mechanical properties as virgin carbon fibers. 

The recovery of carbon fibers from end-of-life composite materials or prepreg waste can be achieved through pyrolysis, solvolysis or fluidized bed processes [5]. Pyrolysis is the most widespread recycling method, and, in this process, composites are heated at high temperatures in the absence of oxygen to decompose the resin [6,7,8]. This leads, on one hand, to pyrolysis gases and residual oil coming from the resin and, on the other hand, to carbon fibers. Pyrolyzed recycled carbon fibers (rCFs) are often considered to be low-quality carbon fibers, but ELG Carbon Fibre (now Gen 2 Carbon) states that their pyrolyzed carbon fiber typically retains at least 90% of its tensile strength with no change in modulus [9]. By means of solvolysis, it is also possible to obtain virgin carbon fiber quality [10]. So, recycled carbon fibers are very interesting for making short-fiber composites using manufacturing processes such as bulk molding compounds (BMC) or sheet molding compounds (SMC) in combination with thermoset resins or injection molding in combination with a thermoplastic polymer [5,11,12]. Another option is to convert the recycled short fibers into a nonwoven and obtain new composites through, for example, resin infusion or compression molding [5,11,13,14,15,16].

Although the mechanical properties of pyrolyzed or solvolyzed carbon fibers are almost similar to virgin chopped fibers, they do not contain a sizing anymore. Sizing is a thin coating applied to the surface of the fibers before they are incorporated into the composite material. It plays a crucial role in the performance and properties of composite materials by improving the adhesion between fibers and the matrix, protecting fibers, and facilitating the manufacturing process. 

Sizing formulations are typically proprietary and developed by manufacturers based on their specific requirements and processing conditions. However, it is known that the main components of a sizing formulation are typically a film former, lubricants and additives such as coupling agents (for glass fibers) and processing additives dispersed in water [17,18,19,20]. Film formers are essential components of sizing agents as they are responsible for creating a thin, uniform film on the surface of the fibers. Common types of film formers are epoxy resins, polyurethanes, polyvinyl acetates polyesters and polyolefins [18]. The film former should adhere well to the fiber surface and promote good bonding with the matrix material. For example, Yan et al. developed a sizing agent that forms a strong interface between carbon fibers and the PA6 matrix. It was found that the average interlaminar shear strength, tensile strength and flexural strength increase by 14.2%, 10.5% and 20.0%, respectively, after the treatment of desized carbon fibers with their sizing agent due to the effective stress transfer across the interface [20].

For virgin carbon fibers, the sizing is applied during the manufacturing process and is thus applied on continuous fibers. However, after pyrolysis, the sizing is removed from the carbon fibers, meaning that a resizing step is recommended to improve their compatibility with a matrix material in composite manufacturing. Thermoplastic composites could especially benefit from this resizing as they usually adhere less well compared to thermoset composites [21].

Because these recycled carbon fibers are discontinuous, researchers are mainly using a batch-type dipping process to resize the fibers and use them for injection molding [17,21]. However, from a processing and economical point of view, a continuous resizing process is more favorable.

Therefore, in this paper, we focused on another method to pretreat recycled short carbon fibers to make them more compatible with a polyamide 6 (PA6) and a polypropylene (PP) matrix. Instead of using recycled loose carbon fibers, we selected nonwovens made from pyrolyzed carbon fibers which, thus, do not contain a sizing anymore. These nonwovens were treated with different sizing formulations (also referred to here as resizing) in order to prepare recycled carbon/PA6 and carbon/PP composites with improved mechanical properties compared to non-resized composites. The formulations were sprayed on carbon nonwovens using a discontinuous process at lab scale, but this process can be easily scaled up to a continuous roll-to-roll process commonly used in the textile finishing industry. Because the exact composition of the sizing formulations utilized in industry is secret, we selected and prepared our own formulations based on discussions with chemical suppliers. After the ‘resizing’ of the carbon nonwovens, carbon/PA6 and carbon/PP composites were prepared by compression molding. The tensile properties and interlaminar shear strength of the developed composites were measured to determine the effect of sizing, and the results were compared to the results of carbon/PA6 and carbon/PP composites without sizing.

## 2. Materials and Methods

### 2.1. Materials

Nonwovens of 200 g/m^2^ with a thickness of about 3 mm and prepared from pyrolyzed carbon fibers were supplied by Gen 2 Carbon at an A5 size. The PA6 was purchased from Nyobe (Kruisem, Belgium), and the PP from TotalEnergies (Feluy, Belgium). For making the sizing formulations, film formers Hydrosize PA 845H and Hydrosize PP2-01 were obtained from Michelman Int’l Belgium SRL (Aubange, Belgium), coupling agent Silquest 1524 and wetting agent Coatosil 1211C were supplied by Grolman Benelux BV (De Goor, The Netherlands), and Tego Airex 902 W from Evonik (Essen, Germany) was selected as a defoamer.

### 2.2. Preparing Sizing Formulations

Sizing formulations were prepared by mixing several components in water. An overview of the different sizing formulations is presented in Table 1. The sizing formulations containing Hydrosize PA845H were designed to be compatible with a PA matrix. Hydrosize PA845H is a PA dispersion with a solid content of 30 w% and is recommended by Michelman to be used for fiber-reinforced PA composites [22]. This film former dispersion was further diluted with deionized water to obtain sizing film formulations at two different concentrations (Forms 1 and 2). To investigate whether additives could improve the performance of the sizing, formulations containing a coupling agent (Silquest A-1524), a wetting agent (CoatOSil 1211C) and a defoamer (Tego Airex 902W) were also prepared (Forms 3 and 4). Silquest A-1524 or gamma-ureidopropyltrimethoxysilane is a 100% active ureidosilane which can be used to promote adhesion between polymers and reinforcements. This silane provides nitrogen reactivity without the strong basic characteristics of typical aminosilanes [23]. CoatOSil 1211C additive enables the wetting of waterborne systems on common and hard-to-wet substrates [24]. TEGO^®^ Airex 902 W is a deaerator emulsion based on polyether siloxane technology [25]. CoatOSil 1211C and TEGO^®^ Airex 902 W are both recommended for spray applications. 

For the PP composites, two sizing formulations were prepared by diluting Hydrosize PP201 (Form 5 and 6). This is a PP dispersion with a solid content of 40 w% and is recommended by Michelman to be used for fiber-reinforced PP composites [26]. In this case, no extra additives were added. 

**Figure 1:**
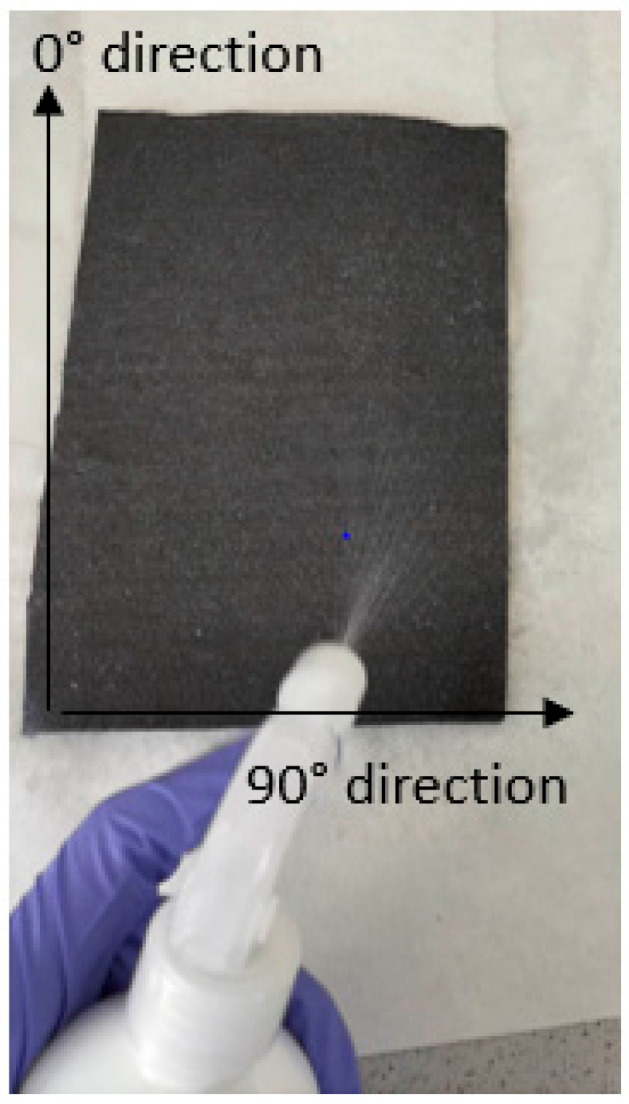
Applying the sizing formulation.

These formulations were applied along both sides of the nonwoven with a hand sprayer until the surface of the nonwoven was saturated (see Figure 1). The figure also shows 2 directions to indicate in which direction the composite samples made from these nonwovens were tested. After spraying, the nonwovens were dried in a convection furnace (Binder, Model 115 from BINDER GmbH, Tuttlingen, Germany) at 100 °C for 10 min. The amount of sizing was determined by measuring the weight difference before and after applying the sizing. 

### 2.3. Preparing Composites

To prepare the composites, polymer (PA6 and PP) pellets were put on top of the nonwoven to obtain a fiber volume content of 20%, and 2 layers of the recycled carbon nonwovens containing 80v% of PA6 or PP were stacked. These materials were pressed using a Fontijne press model Lab Econ 600 (Rotterdam, The Netherlands) at 40 kN for 15 min at temperatures of 240 °C and 190 °C, respectively, with a cooling speed of 15 °C/min. This process was repeated to obtain composites with different fiber sizings and a reference composite containing no sizing. The composites had a thickness of 1 mm. 

### 2.4. Characterization

A JSM-7600F FEG-Scanning Electron Microscope from JEOL (Akishima, Japan) with a resolution of 2 nm was used for scanning electron microscopy (SEM) imaging under a high vacuum to visualize the sizing on the carbon fibers and to investigate how the fibers are embedded in the matrix. A 4 nm thick platina/palladium layer was sputtered on the samples to avoid electrical charging. 

The tensile properties and the interlaminar shear strength were determined using a 20 kN RetroLine tensile tester from Zwick/Roell (Venlo, The Netherlands) on three and five samples in each direction, respectively.

## 3. Results

### 3.1. Post-Treatment (‘Resizing’) of the Recycled Carbon Nonwovens

After the sizing was applied on the carbon nonwovens, the amount of sizing was determined by weighing the samples before and after treating the nonwovens. The results presented in Table 1 are in line with what was expected, namely, the higher the concentration of the film former in the sizing formulation, the higher the amount of sizing on the carbon fiber (e.g., Form 1 vs. Form 2, and Form 5 vs. Form 6). It can be observed that there is some variation in the weight percentage because the sizing was manually sprayed, and the nonwoven does not always absorb the same amount of the formulation (Form 1 vs. Form 3). Also, the sizing content is higher for the Hydrosize PP201 sizing formulation compared to the Hydrosize PA845H-based formulation. The main reason for this is that the received Hydrosize PP201 dispersion has a higher solid content (around 40%) than the received Hydrosize PA845H dispersion (around 30%). 

**Figure 2:**
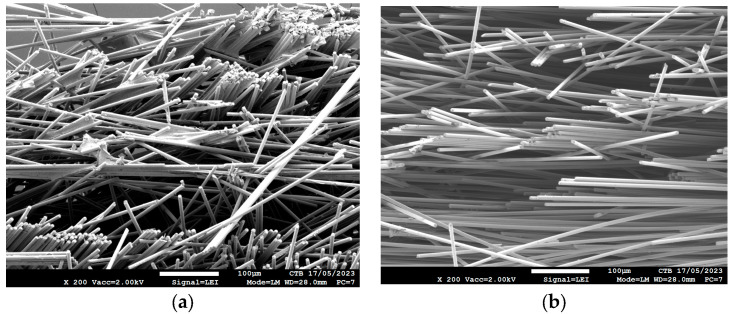
SEM images of treated carbon nonwoven: (**a**) bottom of sample treated with Form 1; (**b**) middle of sample treated with Form 1.

The uniformity of the applied sizing was investigated with SEM. An SEM picture was taken at the surface (Figure 2a) and in the middle (Figure 2b) of the carbon fiber nonwoven treated with sizing formulation 1.

In Figure 2a, it can be seen that when the fibers are grouped in a bundle, the sizing glues the fibers in the bundle together. This makes the nonwoven a bit stronger and easier to handle during composite manufacturing because fewer fibers are coming loose. The SEM analysis also revealed that most of the sizing is present on the surface and not in the bulk of the nonwoven (Figure 2b), meaning that the nonwoven is not fully impregnated. It seems that not even at the surface is every fiber completely covered. Therefore, the sizing is not homogeneously applied on the recycled carbon fibers.

**Figure 3:**
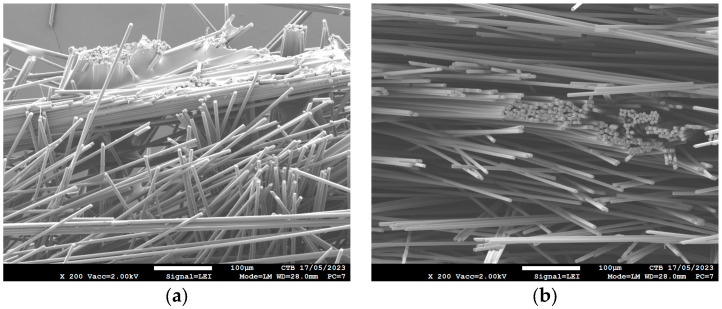
SEM images of treated carbon nonwoven: (**a**) bottom of sample treated with Form 5; (**b**) middle of sample treated with Form 5.

Even for the sample that contains the highest amount of sizing (Form 5) no sizing can be seen in the middle (Figure 3b). More sizing only leads to more coating on the surface of the nonwoven (Figure 3a).

### 3.2. Composites

To know if the pretreatment of the recycled carbon fiber nonwoven with a sizing formulation, although not homogenously applied, could still have a positive influence on the mechanical properties, composites with and without sizing were prepared by compression molding, and the tensile properties and interlaminar shear strength were determined. 

**Figure 4:**
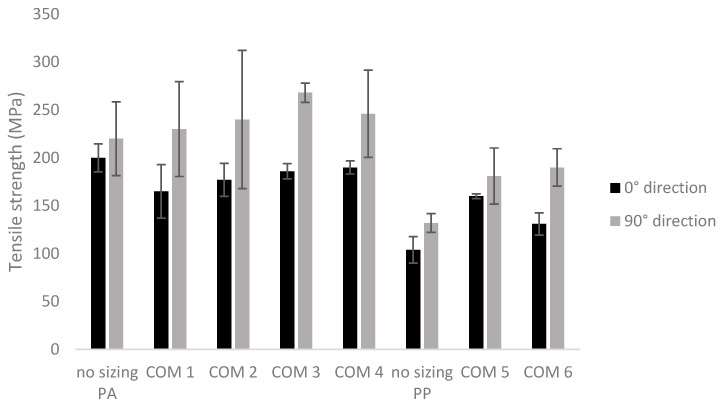
Tensile strength of carbon/PA6 and carbon/PP composites containing different fiber sizings. In black are the results obtained in the 0° direction and in gray are the results obtained in the 90° direction of the composite plate.

Figure 4 shows the effect of the treatment on the tensile strength of the composites with a PA6 and PP matrix. The tensile strength was determined in two directions, namely in the 0° and 90° directions, as indicated in Figure 1.

The sample “no sizing PA” is the PA6 composite without sizing, and the sample “no sizing PP” is the PP composite without sizing. The numbers of the other composites correspond to the respective numbers of the sizing formulations (Form 1–6). Composite COM 1 was thus prepared from nonwovens treated with sizing formulation Form 1. Also, COM 1 to 4 are composites with a PA6 matrix, and COM 5 and 6 are the PP carbon fiber composites.

The graph shows that the tensile strength is higher in the 90° direction than in the 0° direction for all the composites, meaning that the composites are not isotropic. When looking at the PA6 composites, it seems that the pretreatment increases the tensile strength in the 90° direction with the highest average values for COM 3 (268 MPa) and 4 (246 MPa) and the lowest for the non-resized reference sample (220 MPa). On the other hand, no improvement is observed in the 0° direction (200 MPa for no sizing PA, 165 MPa for COM 1, 177 MPa for COM 2, 186 MPa for COM 3 and 190 MPa for COM 4). However, the quite high standard deviations for most of these composites indicates that these differences are not significant and indicates that the resizing does not have a high impact on the tensile properties of the PA6 composites. When looking at the PP matrix, it can be observed that the sizing improves the tensile strength in both directions in contrast to the results of the PA6 composites. When looking at the two directions, COM 5, in which the carbon reinforcement is pretreated with a higher concentration of Hydrosize PP201, has the highest overall tensile strength. This is 54% (160 MPa vs. 104 MPa) more in the 0° direction and 37% (181 MPa vs. 132 MPa) more in the 90° direction than the reference composite (no-sizing PP sample). 

**Figure 5:**
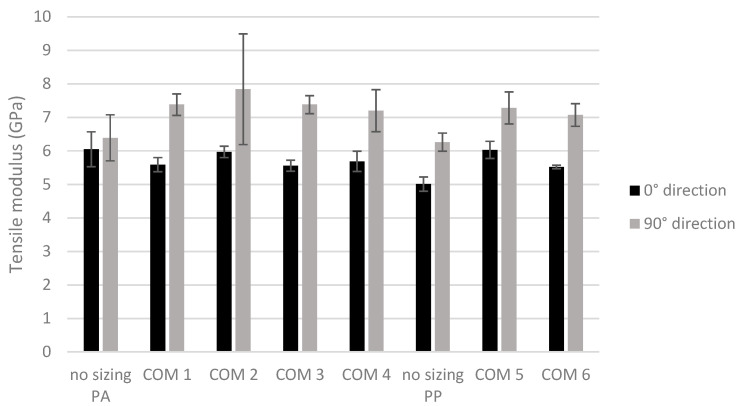
Tensile modulus of carbon/PA6 and carbon/PP composites containing different fiber sizings. In black are the results obtained in the 0° direction and in gray are the results obtained in the 90° direction of the composite plate.

The tensile modulus of the composites is presented in Figure 5. The results are in line with the results of the tensile strength. On average, higher tensile moduli were found for the ‘resized’ PA6 composites in the 90° direction, but not in the 0° direction. The average tensile modulus of the PP composites is higher after pretreatment in both directions compared to the non-resized composites. This indicates that resizing of the recycled carbon fiber nonwoven can improve the tensile properties of PP carbon composites.

**Figure 6:**
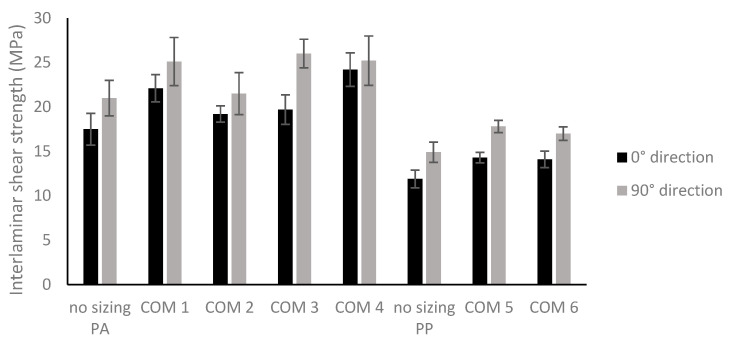
Interlaminar shear strength of carbon/PA6 and carbon/PP composites containing different fiber sizings. In black are the results obtained in the 0° direction and in gray are the results obtained in the 90° direction of the composite plate.

Figure 6 presents the average interlaminar shear strengths and the standard deviations. It can be seen from the graph that, in general, the ILLS value can be increased by a pretreatment step of the carbon nonwoven for both directions. Also, the ILSS values are higher for the PA6 composites than for the PP composites with and without resizing. For the PA6 composites, there is no clear effect of the film former concentration in the sizing formulation (COM 1: 22.1 MPa for 0°; 25.1 MPa for 90° vs. COM 2: 19.2 MPa for 0°; 21.5 MPa for 90° or COM 3: 19.7 MPa for 0°; 26 MPa for 90° vs. COM 4: 24.2 MPa for 0°; 25.2 MPa for 90°) on the interlaminar shear strength or adding extra additives (COM 1 vs. COM 3 and COM 2 vs. COM 4). For the PP composites, there is no difference between 5% (COM 6: 14.1 MPa for 0°; 17.0 MPa for 90°) and 10% (COM 5: 14.3 MPa for 0°; 17.8 MPa for 90°) of film former present on the carbon fiber for the interlaminar shear strength, but both treatments have a positive effect on the shear strength, as the average values of the non-sized PP composites in the 0° and 90° directions are 11.9 MPa and 14.9 MPa, respectively.

To confirm that the sizing indeed results in a significant improvement in the mechanical properties, a statistical analysis was performed via one-way ANOVA and Dunnett’s test with a significance level α of 0.05. For the results of PA6, only the ILSS results have a significant difference between the reference composite without sizing and the composite COM 1 for both directions. In the 0° direction, this is also the case for COM 4, and in the 90° direction, a significant improvement is also found for COM 3. For the PP composites with sizing, the Dunnett’s tests confirm that the improvement due to the sizing is significant, except for the tensile modulus of COM 6 in the 90° direction. The ANOVA and Dunnett’s analyses on the ILSS results of the PP sizing in the 0° direction and 90° direction can be seen in Table 2 and Table 3, respectively. The ANOVA and Dunnett’s analyses of the tensile results of the PP composites can be found in the Supplementary Materials (Tables S1–S12), as can the results of the PA6 composites for tensile properties and interlaminar shear strength.

**Figure 7:**
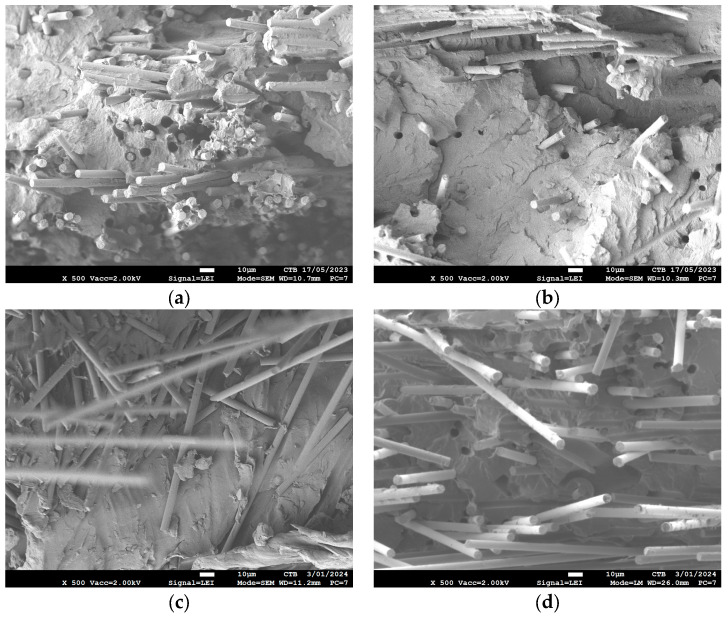
SEM images of the cross section of (**a**) non-pretreated PA6 composite (no-sizing PA), (**b**) pretreated PA6 composite (COM 4), (**c**) non-pretreated PP composite (no-sizing PP) and (**d**) pretreated PP composite (COM 6).

An SEM analysis of the composite’s cross sections was also performed to investigate the effect of the fiber treatment on the adhesion between the fiber and polymer matrix (Figure 7). Figure 7a is the SEM image of the non-resized PA6 composite (no-sizing PA) and 7b is a SEM image of the resized PA6 composite with the highest ILLS strength in the 0° direction (COM 4). In general, the SEM images are similar. Only in some small places can more cracks be found in the untreated composites, which could indicate slightly less fiber–matrix adhesion. Figure 6c is a SEM image of the PP composite without sizing. It can be observed that the fibers are just covered with some matrix, but there is no bonding, resulting in poor fiber–matrix adhesion. This is much better after pretreatment, and it can even be observed that pulled-out fibers during breaking still contain some polymer matrix (Figure 7d). So, the SEM analysis confirms the tensile and interlaminar shear test results. Only small differences for the PA6 composites, but clear differences for the PP composites, were found.

## 4. Discussion

The use of recycled carbon fibers contributes to the circular economy by extending the life cycle of carbon fiber-reinforced materials and reducing the reliance on newly produced fibers. However, it is important that the fiber–matrix adhesion is optimal to obtain the best composite performance. Here, our focus was to improve the tensile properties and interlaminar shear strength of PA6 and PP composites containing pyrolyzed carbon fibers by carrying out a resizing step of these fibers. Instead of trying to treat individual fibers, the sizing formulation was applied on nonwoven reinforcements by spraying. The advantages of this approach over working with loose carbon fibers which have a low bulk density are that the application could be, in principle, performed at industry scale in a continuous way, no rinsing step is required, and there is also less risk of carbon dust flying around in the workplace because the fibers are already processed into a nonwoven.

Although the SEM images reveal that not all the fibers were coated, it is still possible to increase the mechanical properties of the PP composites. For the PA6 composites, the average results of the tensile and interlaminar shear strength properties also indicate a positive effect, but due the high standard deviations, it is difficult to draw conclusions on the impact of the sizing and on the influence of the sizing concentration or the addition of extra additives to the formulation. The reason for the high standard deviations is probably twofold. Firstly, due to the non-uniform distribution of the sizing, the amount of sizing was not the same for each tested composite sample. Some samples were more/better resized than others, resulting in different mechanical properties. Optimizing the spraying process will probably reduce the standard deviation. Secondly, not only does the application method cause a high standard deviation, but also, the nonwoven substrate is an important factor, because some regions are more fiber-rich than others, and this also has an impact on the mechanical properties. This is especially true for the tensile properties because these are mostly dependent on the fiber orientation [27]. For the interlaminar shear strength, which is more focused on the fiber–matrix adhesion, it seems that the resizing has a positive effect because some of the results were found to be significant (e.g., COM1, 3 and 4). This means that the sizing is compatible with the matrix and can enhance some properties, although the effect is probably not high enough to justify an extra processing step. This can probably be explained by the fact that PA6 has sufficient functionality and that pyrolyzed carbon fibers also have some functional groups present on their surface to make interaction possible with a PA6 matrix [28]. To further clarify the impact of the PA sizing, it should be preferably applied on non-sized UD carbon fibers, and afterwards, the mechanical properties should be compared. However, as the focus in our research lies on the pretreatment of recycled carbon nonwovens using a spray application process, we have not investigated this. 

For the PP, significant improvements in its mechanical properties are obtained. 

This means that the treatment is more effective than the treatment for the PA6 composites. The reason for this is that the inert PP matrix cannot form a good bond with the carbon fibers, resulting in poor fiber–matrix adhesion. A common way to improve the interfacial bonding of a PP matrix with carbon fibers is adding maleic anhydride-grafted-polypropylene (MAPP) as a compatibilizer [29]. The film former Hydrosize PP201 used to pretreat the carbon nonwoven in this research is a non-ionic malleated polypropylene dispersion, and MA increases the polarity of the PP, which leads to better adhesion with the fibers.

Further, as the properties are increased in both directions and for every tested sample, it is supposed that the coating is further distributed in the nonwoven during composite manufacturing and could interact well with the PP matrix due to the relatively long processing time (15 min), high pressure (40 kN) and temperature (190 °C) of the composite press. This maybe explains why more significant results were obtained with this film former compared to the research of Palola et al. [21]. These researchers used the same film former to resize rCFs and used these fibers in injection molding with a PP matrix, but they did not observe significant improvement the mechanical properties after resizing.

However, our findings show that for preparing more qualitative recycled carbon PP composites, applying a precoat on the nonwoven by spraying is a promising approach. For PA6 composites this has not been proven so far, further research will focus on optimizing the application process in order to improve the impregnation of the nonwovens by looking at other application methods, such as padding or an automatic controlled spray system, and by looking for dispersions with a lower particle size to investigate whether these could go deeper into the nonwoven. 

Also, extra characterization of the PP composites (e.g., bending and compression tests) will be performed to investigate whether these properties are improved as well. 

## 5. Conclusions

The aim of this study was to investigate whether the mechanical properties of carbon PA6 and PP composites containing a nonwoven reinforcement made from pyrolyzed carbon fibers could be improved by a resizing step. The sizing was applied using a spray process, which is a fast and easy application process. The SEM results revealed that the coating was not uniformly applied on the fibers. Regarding the mechanical properties, no significant improvements in the tensile modulus and strength could be obtained for the PA6 composites, as shown by the Dunnett’s test, while this was the case for the PP composites. A more than 50% increase in tensile strength can be achieved in the 0° direction and a more than 35% increase in tensile strength can be achieved in the 90° direction of the PP composite plate compared to a non-resized reference PP composite. The interlaminar shear strength could be improved for both composites, with a maximum average value of 26 MPa for the resized PA6 compared to 21 MPa for the non-sized reference and a maximum of 17.8 MPa for the pretreated PP composite compared to 14.9 MPa when not pretreated. So, as a final conclusion, this resizing approach seems to be promising to improve the performance of recycled carbon fiber PP composites, but it is not really necessary to resize the nonwovens when they will be combined with a PA6 matrix.

## Figures and Tables

**Table 1:** Sizing formulations.

Formulation	Water (w%)	Hydrosize PA 845H (w%)	Hydrosize PP201 (w%)	Silquest A-1524 (w%)	CoatOSil 1211C (w%)	Tego Airex 902W (w%)	Amount Sizing (w%)
Form 1	90	10	0	0	0	0	4.7
Form 2	95	5	0	0	0	0	3
Form 3	89.85	10	0	0.05	0.05	0.05	3.5
Form 4	94.85	5	0	0.05	0.05	0.05	2.5
Form 5	90	0	10	0	0	0	10
Form 6	95	0	5	0	0	0	5

**Table 2:** ANOVA and Dunnett’s test on ILSS results of PP composites without (REF) and with sizing (COM 5 and COM 6) in 0° direction.

ANOVA						
Source of Variation	SS	df	MS	F	*p*-Value	F Crit
Between Groups	17.669	2	8.835	14.272	0.001	3.885
Within Groups	7.428	12	0.619			
Total	25.097	14				
DUNNETT						
	Absolute Mean Difference	Dunnett’s value				
REF vs. COM 5	2.36	1.24				
REF vs. COM 6	2.24	1.24				

**Table 3:** ANOVA and Dunnett’s test on ILSS results of PP composites without (REF) and with sizing (COM 5 and COM 6) in 90° direction.

ANOVA						
Source of Variation	SS	df	MS	F	*p*-Value	F Crit
Between Groups	21.561	2	10.781	6.490	0.012	3.885
Within Groups	19.932	12	1.661			
Total	41.493	14				
DUNNETT						
	Absolute Mean Difference	Dunnett’s value				
REF vs. COM 5	2.82	2.04				
REF vs. COM 6	2.12	2.04				

## Data Availability

The data presented in this study are available on request from the corresponding author.

## Supplementary Materials

The following supporting information can be downloaded at: https://www.mdpi.com/article/10.3390/polym16040561/s1, Table S1: ANOVA on tensile strength of PA composites in 0° direction; Table S2: ANOVA on tensile strength of PA composites in 90° direction; Table S3: ANOVA and Dunnett’s test on tensile modulus of PA composites without (REF) and with sizing (COM 1–COM 4) in 0° direction; Table S4: ANOVA on tensile modulus of PA composites in 90° direction; Table S5: ANOVA and Dunnett’s test on ILSS of PA composites without (REF) and with sizing (COM 1–COM 4) in 0° direction; Table S6: ANOVA and Dunnett’s test on ILSS of PA composites without (REF) and with sizing (COM 1–COM 4) in 90° direction; Table S7: ANOVA and Dunnett’s test on tensile strength of PP composites without (REF) and with sizing (COM 5–COM 6) in 0° direction; Table S8: ANOVA and Dunnett’s test on tensile strength of PP composites without (REF) and with sizing (COM 5–COM 6) in 90° direction; Table S9: ANOVA and Dunnett’s test on tensile modulus of PP composites without (REF) and with sizing (COM 5–COM 6) in 0° direction; Table S10: ANOVA and Dunnett’s test on tensile modulus of PP composites without (REF) and with sizing (COM 5–COM 6) in 90° direction, Table S11: ANOVA and Dunnett’s test on ILSS of PP composites without (REF) and with sizing (COM 5–COM 6) in 0° direction; Table S12: ANOVA and Dunnett’s test on ILSS of PP composites without (REF) and with sizing (COM 5–COM 6) in 90° direction.
