# Supplementary material for: Pretreating Recycled Carbon Fiber Nonwoven with a Sizing Formulation to Improve the Performance of Thermoplastic Recycled Fiber-Reinforced Composites
Source: Polymers (Basel). 2024 Feb 19;16(4):561. doi: 10.3390/polym16040561 (PMC10893380; doi:10.3390/polym16040561)
Supplement: Supplementary file 1 [file polymers-16-00561-s001.zip › polymers-2844031-supplementary.pdf]

Article

Improving the mechanical properties of recycled carbon fiber nonwoven thermoplastic composites by pretreating the nonwoven with a sizing formulation

Supporting information

Table S1: ANOVA on tensile strength of PA composites in 0° direction

ANOVA

Source of Variation	SS	df	MS	F	P-value	F crit
Between Groups	3407.613	4	851.9033	2.442059	0.078535	2.8401
Within Groups	7325.771	21	348.8463			
Total	10733.38	25				

Table S2: ANOVA on tensile strength of PA composites in 90° direction

ANOVA

Source of Variation	SS	df	MS	F	P-value	F crit
Between Groups	6118.864	4	1529.716	0.70968	0.596446	2.964708
Within Groups	36643.5	17	2155.5			
Total	42762.36	21				

Table S3: ANOVA and Dunnett's test on tensile modulus of PA composites without (REF) and with sizing (COM 1 - COM 4) in 0° direction

ANOVA

Source of Variation	SS	df	MS	F	P-value	F crit
Between Groups	978484.5604	4	244621.1	3.784838	0.018063	2.8401
Within Groups	1357269.286	21	64631.87			
Total	2335753.846	25				

DUNNETT

	Absolute Mean Difference	Dunnett's value
REF vs COM 1	455.71	463.145707
REF vs COM 2	77.14	463.145707
REF vs COM 3	488.00	490.1473446
REF vs COM 4	362.50	512.6083629

Table S4: ANOVA on tensile modulus of PA composites in 90° direction

ANOVA

Source of Variation	SS	df	MS	F	P-value	F crit
Between Groups	5790353.03	4	1447588	1.991272	0.141606	2.964708
Within Groups	12358433.33	17	726966.7			
Total	18148786.36	21				

Table S5: ANOVA and Dunnett's test on ILSS of PA composites without (REF) and with sizing (COM 1 - COM 4) in 0° direction

ANOVA

Source of Variation	SS	df	MS	F	P-value	F crit
Between Groups	209.300544	4	52.32514	16.35548	3.17E-06	2.8401
Within Groups	67.18407143	21	3.199241			
Total	276.4846154	25				

DUNNETT

	Absolute Mean Difference	Dunnett's value
REF vs COM 1	4.89	2.959680056
REF vs COM 2	2.01	2.764929358
REF vs COM 3	2.51	2.764929358
REF vs COM 4	8.03	2.764929358

Table S6: ANOVA and Dunnett's test on ILSS of PA composites without (REF) and with sizing (COM 1 - COM 4) in 90° direction

ANOVA

Source of Variation	SS	df	MS	F	P-value	F crit
Between Groups	99.69636813	4	24.92409	5.967475	0.002272	2.8401
Within Groups	87.70978571	21	4.176656			
Total	187.4061538	25				

DUNNETT

	Absolute Mean Difference	Dunnett's value
REF vs COM 1	4.01	3.159183885
REF vs COM 2	0.39	3.159183885
REF vs COM 3	4.90	3.381704313
REF vs COM 4	3.09	3.159183885

Table S7: ANOVA and Dunnett's test on tensile strength of PP composites without (REF) and with sizing (COM 5 - COM 6) in 0° direction

ANOVA

Source of Variation	SS	df	MS	F	P-value	F crit
Between Groups	4637.12	2	2318.56	21.43639	0.00185	5.143253
Within Groups	648.96	6	108.16			
Total	5286.08	8				

DUNNETT

	Absolute Mean Difference	Dunnett's value
REF vs COM 5	55.60	24.311349
REF vs COM 6	27.60	24.311349

Table S8: ANOVA and Dunnett's test on tensile strength of PP composites without (REF) and with sizing (COM 5 - COM 6) in 90° direction

ANOVA

Source of Variation	SS	df	MS	F	P-value	F crit
Between Groups	5900.222222	2	2950.111	6.654386	0.030005	5.143253
Within Groups	2660	6	443.3333			
Total	8560.222222	8				

DUNNETT

	Absolute Mean Difference	Dunnett's value
REF vs COM 5	49.67	49.21992092
REF vs COM 6	58.00	49.21992092

Table S9: ANOVA and Dunnett's test on tensile modulus of PP composites without (REF) and with sizing (COM 5 - COM 6) in 0° direction

ANOVA

Source of Variation	SS	df	MS	F	P-value	F crit
Between Groups	1550422.222	2	775211.1	20.73373	0.00202	5.143253
Within Groups	224333.3333	6	37388.89			
Total	1774755.556	8				

DUNNETT

	Absolute Mean Difference	Dunnett's value
REF vs COM 5	1016.67	452.0089124
REF vs COM 6	506.67	452.0089124

Table S10:ANOVA and Dunnett's test on tensile modulus of PP composites without (REF) and with sizing (COM 5 - COM 6) in 90° direction

ANOVA

Source of Variation	SS	df	MS	F	P-value	F crit
Between Groups	1732422.222	2	866211.1	6.266801	0.033929	5.143253
Within Groups	829333.3333	6	138222.2			
Total	2561755.556	8				

DUNNETT

	Absolute Mean Difference	Dunnett's value
REF vs COM 5	1016.67	869.0898021
REF vs COM 6	810.00	869.0898021

Table S11:ANOVA and Dunnett's test on ILSS of PP composites without (REF) and with sizing (COM 5 - COM 6) in 0° direction

ANOVA

Source of Variation	SS	df	MS	F	P-value	F crit
Between Groups	17.669	2	8.835	14.272	0.001	3.885
Within Groups	7.428	12	0.619			
Total	25.097	14				

DUNNETT

	Absolute Mean Difference	Dunnett's value
REF vs COM 5	2.36	1.24
REF vs COM 6	2.24	1.24

Table S12: ANOVA and Dunnett's test on ILSS of PP composites without (REF) and with sizing (COM 5 - COM 6) in 90° direction

ANOVA

Source of Variation	SS	df	MS	F	P-value	F crit
Between Groups	21.561	2	10.781	6.490	0.012	3.885
Within Groups	19.932	12	1.661			
Total	41.493	14				

DUNNETT

	Absolute Mean Difference	Dunnett's value
REF vs COM 5	2.82	2.04
REF vs COM 6	2.12	2.04
